# Geopolymer-Derived Aluminosilicate Matrix Composites Reinforced with Continuous Nickel-Coated Carbon Fibers: Tensile Properties and Thermo-Oxidative Behavior

**DOI:** 10.3390/polym18192448

**Published:** 2026-10-08

**Authors:** Michal Jasiczek, Marcelina Bobrowska, Arnold Jedral

**Affiliations:** 1 GE Aerospace, Al. Krakowska 110/114, 02-256 Warsaw, Poland; 2 Łukasiewicz Research Network–Institute of Aviation, Al. Krakowska 110/114, 02-256 Warsaw, Poland

**Keywords:** geopolymer composite, aluminosilicate matrix, electrolytic nickel coating, carbon fiber, continuous-fiber composite, thermo-oxidative behavior, tensile strength

## Abstract

This study aims to evaluate the feasibility of an aluminosilicate geopolymer composite reinforced with continuous nickel-coated carbon fibers and establish its baseline mechanical properties and thermo-oxidative behavior. Unidirectional and balanced cross-ply laminates were fabricated via slurry impregnation, drum winding, and autoclave consolidation. The as-fabricated laminates exhibited a bulk density of 1.94 ± 0.16 g/cm^3^ and an apparent porosity of 23.8 ± 2.5%. For the [0]_8_ laminates, the mean tensile strength was 827.0 MPa at room temperature, 832.1 MPa at 371 °C, 745.7 MPa at 427 °C, and 365.5 MPa at 538 °C. Temperature-dependent failure modes shifted from predominantly longitudinal splitting up to 371 °C, to mixed splitting and fiber-dominated tensile fracture within the gauge section at 427 °C, and finally to brittle, fiber-dominated tensile fracture with no apparent fiber pull-out at 538 °C. Thermo-oxidative studies showed that the net mass loss of the coupons remained below 0.4% after 1000 h at 316 °C but reached approximately 4.7% after 336 h at 371 °C. The sections examined after exposure at 371 °C showed non-uniform filament thinning. These results establish a baseline mechanical and thermo-oxidative characterization for the proposed material system and identify prolonged oxidation as a limitation requiring further evaluation.

## 1. Introduction

The continued rise in operating temperatures in aerospace and propulsion systems has intensified the need for materials that combine low density with reliable performance under thermal and oxidative exposure [1]. Continuous-fiber composites were developed to maximize macroscopic strength and stiffness by aligning the fibers parallel to the direction of applied loads, relying on a cohesive matrix phase to shield the reinforcement and ensure efficient stress transfer. This design principle underlies both polymer-matrix composites (PMCs) and ceramic-matrix composites (CMCs), though in the latter, an appropriately tailored interface may be required to permit debonding and sliding, thereby allowing the fibers to bridge matrix cracks and promote progressive rather than abrupt failure [2]. The combination of efficient load transfer and fiber-mediated damage tolerance makes the choice of continuous-fiber reinforcement central to lightweight, load-bearing composite systems.

Within the landscape of lightweight composite materials, the upper temperature capability of PMCs is governed largely by the thermal stability of the organic matrix. In epoxy systems, thermal softening near the moisture-dependent glass-transition temperature (*T_g_*) reduces the retention of matrix-dominated properties [3]. Representative dry and moisture-conditioned *T_g_* values for standard commercial epoxy systems are summarized in Table 1 [4,5].

Bismaleimide (BMI) and polyimide resins were developed to extend the operational temperature window of PMCs [6]. Commercial carbon-fiber/BMI systems are typically rated for continuous service at 204 °C and short-duration exposure up to 232 °C [7,8]. Temperature-dependent changes in the fiber–matrix interface can reduce composite mechanical properties [9]. In carbon/glass hybrid BMI composites, prolonged thermo-oxidative aging has been associated with matrix degradation, fiber debonding, and reduced interlaminar shear strength [10]. Consequently, the practical temperature capability of a BMI composite depends on its *T_g_*, exposure duration, environmental conditions, and the specific mechanical property that must be retained.

Polyimides have become especially important because they offer high thermal stability, chemical durability, and a favorable strength-to-weight ratio, making them suitable for severe aerospace environments [11]. Their practical value, however, depends not only on nominal temperature capability but also on maintaining performance under application-specific environmental conditions [12]. Oxidative degradation depends on the specific exposure environment, oxygen availability, transport conditions, temperature, and exposure duration [12,13]. PMR-15 serves as a well-characterized aerospace reference system [14,15]. While its glass-transition temperature provides an initial screening metric, long-term durability is heavily governed by time-dependent thermo-oxidative damage. Isothermal air-aging studies at 204–316 °C demonstrated that oxidation progresses from exposed surfaces into the PMR-15 resin and composite specimens, forming an oxidized surface layer that degrades compressive properties, particularly in thinner laminates [16]. Consequently, *T_g_* alone is insufficient to define an operational service limit, as oxygen access and time-dependent thermo-oxidative damage also influence the retained properties.

Research aimed at extending polyimide-composite capabilities has focused on both increasing *T_g_* and improving property retention during prolonged thermo-oxidative exposure. Relative to PMR-15, DMBZ-15 has a higher reported post-cure *T_g_* (414 versus 348 °C); however, after 1000 h in air at 288 °C, its fabric laminate exhibited a larger reduction in compressive strength than the corresponding PMR-15 laminate [17]. RM-1100 is a next-generation polyimide system engineered for continuous service at 343 °C with short excursions up to 371 °C. Although this system has a reported dry *T_g_* of 385 °C, the cited public sources do not provide long-term property-retention data for this material [18,19,20]. Limited research has examined backbone-ethynyl crosslinking; one reported system had a *T_g_* of 486 °C and retained flexural properties during short-duration testing at 400 °C [21].

When the temperature capability of polyimide composites is insufficient, continuous-fiber CMCs offer a higher-temperature alternative for weight-sensitive structures. Their fabrication, however, can require repeated high-temperature densification steps [22], increasing process complexity. Considering the thermal limitations of organic matrices and the processing requirements of CMCs, there is a motivation to investigate alternative inorganic matrix synthesis routes that may simplify processing while retaining resistance to thermal degradation.

One pathway considered here is the geopolymer approach, which enables the formation of inorganic matrices for continuous-fiber composites through comparatively low-temperature hardening [23]. Geopolymerization allows for the synthesis of inorganic aluminosilicate networks via alkali activation and curing at relatively low temperatures. For continuous-fiber composites, this processing strategy provides an alternative to organic matrices and avoids the repeated high-temperature densification used in some CMC fabrication routes [22,24]. The viability of fabricating these matrices via slurry impregnation and prepreg manufacturing has recently been demonstrated for a geopolymer-derived composite reinforced with continuous Inconel 601 fibers [24].

Studies of discontinuous (short) fiber-reinforced geopolymers include construction-oriented systems and a range of reinforcement types [25,26]. Reported functions include control of thermal shrinkage [27] and increased tensile strain capability [28]. Because their reinforcement form and intended function differ from those of continuous-fiber-reinforced laminates, these systems do not provide a direct benchmark. Consequently, the present study focuses on continuous-fiber composites. Table 2 summarizes the most relevant literature and available data on continuous-fiber geopolymer-derived inorganic composites, categorized by key performance metrics (flexural and tensile properties), manufacturing conditions, and environmental exposure prior to testing. These studies primarily report the initial, as-manufactured properties of specific material systems.

Early work on potassium-aluminosilicate/carbon-fabric cross-ply laminates demonstrated that geopolymer-matrix carbon-fiber composites could achieve a tensile strength of 343 MPa at 22 °C [29]. In the same study, however, the flexural strength was shown to drop from 245 MPa at 22 °C to 163 MPa after exposure to air at 400 °C for more than 60 min, indicating reduced flexural performance under the reported exposure conditions [29]. Continuous carbon reinforcement has subsequently been incorporated into both potassium-silicate/thermal-silica/kaolin and metakaolin-based geopolymer formulations [30,31]. For instance, Tran et al. reported a flexural strength of approximately 571 MPa after curing and drying at 75 °C [30]. Meanwhile, Medri et al. achieved a tensile strength of 136.7 MPa for carbon-fabric laminates following curing at 80 °C and subsequent thermal stabilization at 750 °C in an argon atmosphere [31].

The present study addresses this research gap by combining as-manufactured tensile characterization with observations following prolonged high-temperature exposure in air for continuous nickel-coated carbon-fiber/geopolymer-derived laminates. The material concept is motivated by two related challenges: efficient load transfer between the fibers and matrix and reduced oxygen access to the carbon fibers during high-temperature exposure.

The first design consideration is the preservation of fiber–matrix load transfer during thermal exposure. Long-term structural performance relies on maintaining a stable fiber–matrix interface capable of efficiently transferring mechanical loads into the continuous fibers. Consequently, any degradation of the interfacial strength can severely compromise laminate mechanical properties, even when the fibers remain intact. Regarding uncoated carbon-fiber/geopolymer systems, synchrotron total-scattering analysis of a carbon-fiber/metakaolin geopolymer identified an additional atom-pair correlation that was tentatively assigned to Al–O–C bonding [32]. The reported interphase properties were intermediate between those of the fiber and matrix. However, the thermal stability of this interphase, as well as its effectiveness in preventing oxygen access to the carbon fiber core during prolonged thermal exposure, remains unknown.

A second relevant design consideration concerns the risk of fiber oxidation due to oxygen access to the fibers via porosity or matrix cracking due to thermal mismatch between the fiber and the matrix. In advanced C/SiC systems, oxidation-life models relate oxygen ingress to matrix and seal-coat cracks [33]. Although this mechanistic link serves as a design analogy, it supports evaluating a fiber-level barrier intended to limit oxygen access to the reinforcing phases. Potential microcracking or connected porosity may provide gas-transport paths during thermal exposure. Nitrogen-permeability measurements on heated, sodium-activated metakaolin mortars show that gas transport depends on formulation [34]. An inorganic matrix, therefore, should not be assumed to protect carbon fibers as an effective oxygen barrier without material-specific transport measurements. Furthermore, graphite provides a thermodynamic reference for carbon oxidation. NIST-JANAF data give a standard Gibbs energy of approximately −394.4 kJ mol−1 at 25 °C and −395.6 kJ mol^−1^ at 550 °C for C(graphite) + O_2_(g) → CO_2_(g), confirming a strong thermodynamic driving force over the temperature range considered here [35]. These values describe equilibrium thermodynamics; they do not establish the phase composition of the present fibers, their oxidation kinetics, or the effectiveness of the matrix as an oxygen barrier.

Metallic fiber coatings, particularly nickel coatings, therefore warrant evaluation as potential barriers to oxygen access. Electroless Ni–P coatings have been shown to delay the observed oxidation of carbon fibers during heating in air [36]. Although the coating chemistry differs from the electrolytic nickel coating examined in this study, this result provides experimental motivation for the present material concept. Additionally, geopolymer binders can develop measurable adhesion to selected metallic substrates, although the magnitude depends on binder composition and surface condition [37]. In a recently reported geopolymer-derived aluminosilicate composite reinforced with continuous Inconel 601 fibers, representative fracture surfaces showed little or no apparent fiber pull-out, consistent with load transfer within that material system [24]. These observations provide a rationale for examining a metal-coated reinforcement but do not establish adhesion to the electrolytic nickel coating used here. A nickel coating could limit oxygen access while providing a metallic fiber–matrix contact surface. The present study does not isolate coating efficacy or measure interfacial strength.

The objective of this study is to assess the feasibility of a geopolymer-derived aluminosilicate composite material system reinforced with continuous nickel-coated carbon fibers, with high-temperature polyimide composites serving as a practical industrial benchmark rather than a matched control. The experimental program establishes the as-manufactured physical and tensile properties, documents fracture morphologies after short-duration tensile tests at temperatures up to 538 °C, and records mass change and microstructural evolution during prolonged exposure in air at 316–371 °C. These measurements establish a mechanical and thermo-oxidative baseline for deciding whether further development is warranted for use at temperatures relevant to high-temperature polyimide composites. They do not define a safe service-temperature envelope.

**Table 1:** Selected epoxy, BMI, and polyimide PMC systems and their reported glass-transition or upper-use temperatures.

Matrix Type and Product Designation	Reported *T_g_*	Reported Upper-Use Temperature	Source-Specific Context and Limitations
Epoxy: HexPly 8552 [4]	Neat-resin dry/wet *T_g_*: 200/154 °C	Up to 121 °C	Supplier product data; the *T_g_* values describe neat resin.
Epoxy: CYCOM 977-2 [5]	DMA-onset dry/wet *T_g_*: 170/135 °C	Not stated in the cited source	Supplier product data; the *T_g_* values describe neat resin, and no upper-use temperature is specified.
BMI: HexPly M65 [7]	Neat-resin dry/wet *T_g_*: 300/225 °C	Long-term use up to 204 °C	Supplier product data.
BMI: CYCOM 5250-4 [8]	Neat-resin dry/wet *T_g_*: 300/200 °C	Continuous use to 204 °C; short-duration use to 232 °C	Supplier product data.
Polyimide: PMR-15 [38,39]	*T_g_*: 335 °C by rheological measurement, confirmed by TMA	Not stated as an upper-use temperature in the cited sources	The cited sources concern a woven T650-35/PMR-15 composite under source-specific test conditions; no upper-use temperature independent of reinforcement form and laminate lay-up is established.
Polyimide: DMBZ-15 [17]	Post-cure *T_g_*: 414 °C	Not stated as an upper-use temperature in the cited source	Source-specific aging data at 288 °C include flexural, interlaminar-shear, and compressive properties; the results depend on reinforcement form, laminate lay-up, and the property measured.
Polyimide: RM-1100 [18,19,20]	DMA dry/wet *T_g_*: 385/298 °C	Continuous use to 343 °C; excursion to 371 °C	Supplier product data.
Polyimide: EBPA-2 [21]	Reported *T_g_*: 486 °C	Not stated; short-duration flexural data are reported at 400 °C	Short-duration flexural tests at temperature; no prolonged-aging data for retained properties.

**Table 2:** Summary of processing conditions, mechanical properties, and elevated-temperature aging data for continuous carbon-fiber-reinforced geopolymer-matrix composites.

Properties Characterized	Material System and Reinforcement Form or Lay-Up	Processing or Thermal History	Results	Elevated-Temperature Aging Effects/Upper-Use Temperature
Cross-ply tension	Lyon et al. [29]; potassium-aluminosilicate; carbon-fabric cross-ply, approximately 50–55% fiber volume fraction (FVF)	Three-hour cure at 80 °C	Tensile strength/modulus at 22 °C: 343 MPa/79 GPa. Flexural strength: 245 MPa at 22 °C and 163 MPa after approximately 1 h in air at 400 °C.	Short-duration flexural exposure data only; no prolonged air-aging property retention data.
Unidirectional flexure	Tran et al. [30]; potassium-waterglass/thermal-silica/kaolin; HTS5631 24K UD carbon fiber, approximately 40% FVF	Cure for 5 h and dry for 5 h at 75 °C	Three-point flexural strength/modulus at 22 °C: approximately 571 MPa/64.31 GPa.	No tensile results or prolonged air-aging property retention data.
Balanced-fabric tension	Medri et al. [31]; potassium-polysilicate/metakaolin/fused silica with SiC; ten alternating 0/90 carbon-fabric plies, 35% FVF	Cure at 80 °C; processing stabilization at 750 °C in argon	Room-temperature tensile strength: 136.7 MPa; tensile modulus: 37.4 GPa.	No prolonged air-aging property retention data.
Balanced-fabric tension; datasheet	PyroKarb [40]; proprietary glass-ceramic matrix; 2D 0/90 carbon fabric	Product datasheet; manufacturing cycle not stated	Tensile strength/modulus at 25 °C: 270 MPa/75 GPa; at 350 °C: 250 MPa/73 GPa.	The reported long-term use temperature of 300 °C has no stated duration or atmosphere.

## 2. Materials and Methods

### 2.1. Materials

The reinforcement was a commercially available Grafil 34-700 standard-modulus polyacrylonitrile-based carbon-fiber tow supplied by Mitsubishi Chemical Carbon Fiber (Sacramento, CA, USA) with an electrolytically deposited nickel coating supplied by Electro Fiber Technologies LLC (Schenectady, NY, USA). The nickel content ranged from 40 wt.% to 55 wt.% of the final coated-tow mass. The specified range reflects the average spool-to-spool variability among the fiber-tow spools randomly mixed to fabricate the prepregs. The fibers were supplied as tows and used as received. They served as continuous reinforcement in prepregs subsequently used to manufacture laminates for physical-property measurements, tensile testing, and air-exposure studies. Representative images of the as-received reinforcement are presented in Section 3.1.

The matrix was produced from a precursor system comprising a commercial-grade kaolinite source, together with high-purity, laboratory-grade colloidal silica and silicate and hydroxide sources. No fillers were used in the matrix formulation. Although the exact compositional ratios and detailed slurry formulation are proprietary and therefore not disclosed, the resulting material system consisted of a geopolymer-derived aluminosilicate matrix reinforced with continuous Grafil 34-700 carbon fibers bearing an electrolytically deposited nickel coating.

### 2.2. Composite Fabrication

**Figure 1:**
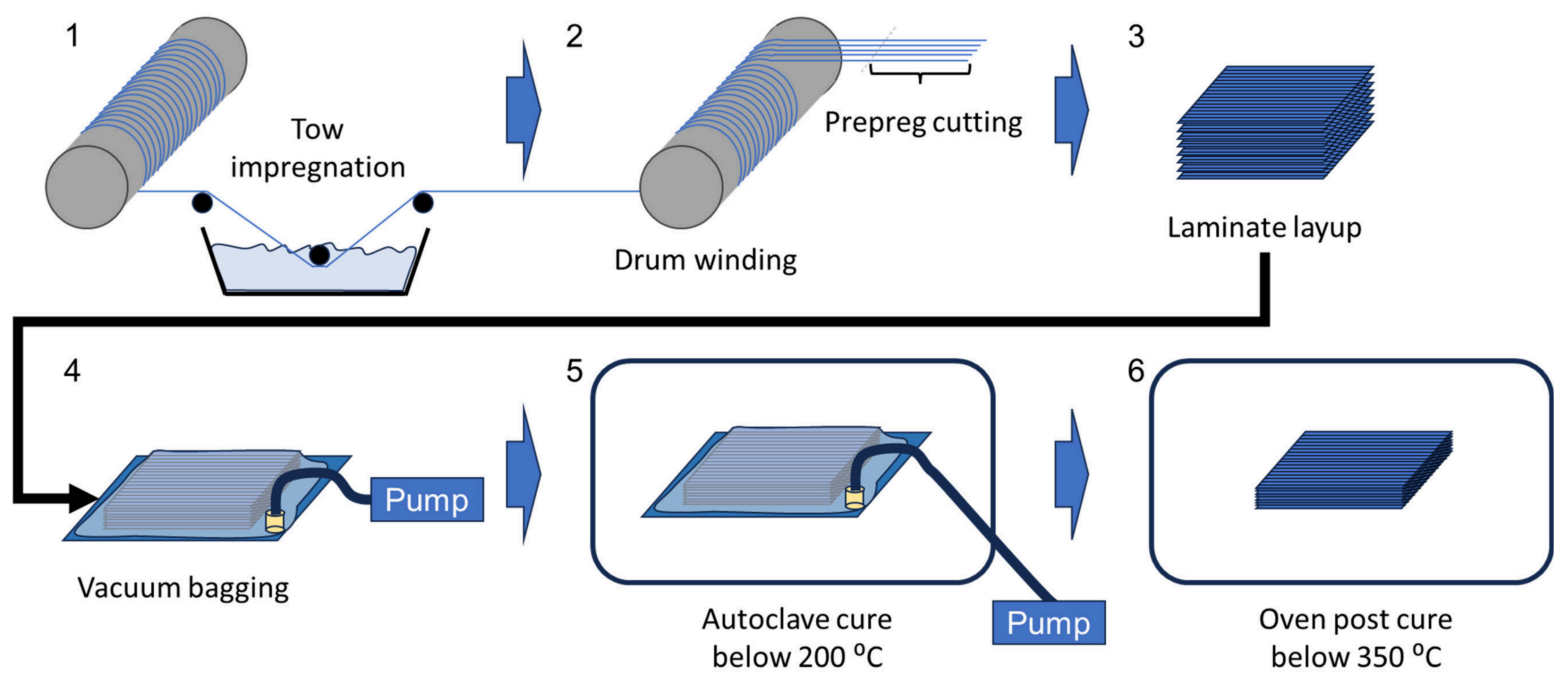
Manufacturing sequence used to produce the composite panels (steps 1–6).

**Figure 2:**
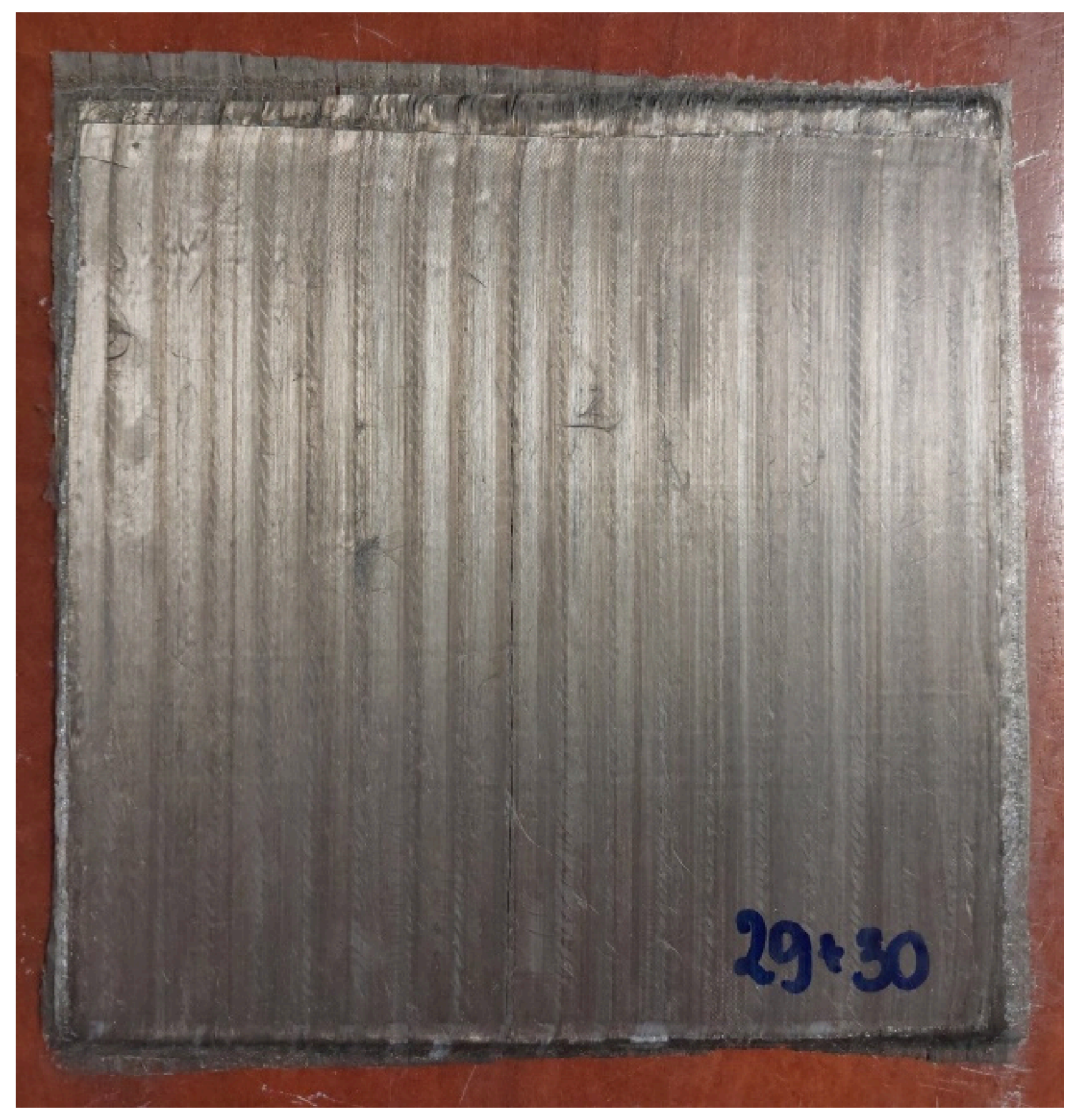
Representative photograph of a fully processed eight-ply [0]_8_ unidirectional composite panel used for machining test specimens and subsequent characterization. Approximate panel dimensions: 200 × 200 mm.

Composite panels were manufactured via a slurry-impregnation and drum-winding prepreg process. The continuous fiber tows were passed through the precursor slurry (step 1), wound onto a drum and cut into prepreg sheets (step 2) for laminate lay-up (step 3). The stacked laminates were vacuum-bagged (step 4) and consolidated in an autoclave (step 5). While the exact cure schedules, tooling configurations, and specific processing parameters are proprietary, the thermal cycle involved curing below 200 °C, followed by a final post-curing stage below 350 °C (step 6) to remove residual volatiles and yield rigid composite panels. A schematic of this fabrication sequence is depicted in Figure 1, and Figure 2 shows a representative eight-ply unidirectional panel. Test coupons were subsequently machined from the fully processed panels to the dimensions specified for each characterization method.

Optical image analysis of polished cross-sections using a KEYENCE VHX-6000 (KEYENCE, Osaka, Japan) and its built-in software gave an estimated reinforcement volume fraction of 45–51% for the laminates. A more rigorous analysis methodology specific to this material system has not yet been established. In particular, the combination of nickel-coated carbon fiber tows and an inorganic geopolymer matrix presents challenges for conventional image-based measurements. These include high sensitivity to specimen preparation, sectioning quality, local tow morphology, and contrast differences among the fiber, coating, and matrix. Accordingly, the values reported here should be regarded as estimates for comparative purposes rather than precise absolute measurements. The composite panels also contained internal porosity, which is characterized in subsequent sections.

### 2.3. Characterization Methods

#### 2.3.1. Bulk Density and Apparent Porosity

Bulk density and apparent porosity were determined via the Archimedes immersion method described in ASTM C20-00 [41], with the modification that non-standard coupons measuring 40 × 40 × 2.1 mm were utilized due to panel-manufacturing constraints. Four unidirectional [0]_8_ specimens, each from a different panel, were evaluated. All masses were recorded using an analytical balance (Mettler Toledo MS105DU) with a readability of 0.00001 g. The reported data represent the mean values of the four specimens. The measured porosity is the apparent liquid-accessible porosity and does not quantify gas permeability.

#### 2.3.2. Tensile Testing

Tensile testing was conducted according to ASTM C1359-18e1 [42]. Unidirectional coupons were tested on an MTS Model 880 servo-hydraulic frame equipped with a 100 kN load cell, and balanced cross-ply coupons were tested on an MTS Landmark 370.10 frame equipped with a 100 kN load cell. The tests were conducted in ISO/IEC 17025- and NADCAP-accredited laboratories for non-metallic materials testing.

Unidirectional [0]_8_ coupons were tested in air at room temperature, 371 °C, 427 °C, and 538 °C. Specimen temperature was monitored with a thermocouple attached to the gauge section, and each specimen was held at the target temperature (±2 °C) for 15–30 min before the load was applied.

Specimens machined from balanced [0/90]_2S_ cross-ply laminates were tested at room temperature to provide a separate mechanical baseline for a laminate with a lower fraction of load-bearing 0° fibers.

The specimen types, dimensions, and sample sizes for each testing condition are summarized in Table 3. For all mechanical tests, the crosshead displacement rate was maintained at 1.3 mm/min. Bonded strain gauges were utilized for the cross-ply specimens, whereas extensometers with a 25.4 mm gauge length were used for the unidirectional coupons. The tensile modulus was determined from the initial linear portion of each stress–strain record, before the first abrupt strain discontinuity or extensometer movement, in accordance with ASTM C1359-18e1 [42]. Three specimens were evaluated for each laminate lay-up and temperature combination, with all three replicates for a given condition machined from the same panel. Accordingly, *n* = 3 captures within-panel specimen variability rather than panel-to-panel or batch-to-batch variation. The resulting means and standard deviations provide descriptive, feasibility-level baselines and are not intended for use as design allowables. Interpretation of the mechanical test results was supported by qualitative, post-test failure-mode observations.

#### 2.3.3. Thermo-Oxidative Exposure

Unidirectional [0]_8_ coupons machined to dimensions of 40 × 40 mm were exposed to air in an air-circulating laboratory oven (SNOL 40/1200LSF21) at nominal temperatures of 316 °C, 343 °C, and 371 °C. A prolonged 1000 h exposure was conducted at 316 °C and 343 °C to evaluate long-term thermo-oxidative effects. The 371 °C exposure was terminated after 336 h to retain specimens for subsequent microstructural examination. The 316 °C condition matched the nominal temperature used in selected hot-air-aging studies of T650-35/PMR-15 polyimide composites, whereas 343 and 371 °C extended the screening into the higher temperature range relevant to advanced polyimide systems summarized in Table 1.

Prior to the initial weighing (t = 0), specimens were dried at 120 °C for 16 h to remove absorbed moisture and volatile species. Intermediate mass measurements were then performed at exposure intervals of approximately 24–200 h using a Mettler Toledo MS105DU analytical balance with a readability of 0.00001 g. Net mass change describes the whole composite because carbon oxidation may decrease mass while oxidation of the metallic coating may increase it. Following exposure, selected specimens were sectioned for microstructural examination to identify thermo-oxidation-induced damage.

#### 2.3.4. Optical and Scanning Electron Microscopy

Optical microscopy was performed using a VHX-6000 optical microscope (Keyence Corporation, Osaka, Japan). Additional microstructural and fracture examinations were carried out using an FEI Nova NanoSEM 450 field-emission scanning electron microscope (SEM) (FEI Company, Hillsboro, OR, USA). Fracture surfaces were observed under low-vacuum conditions using a low-vacuum detector without prior sample preparation. Elemental distributions were examined by energy-dispersive X-ray spectroscopy (EDS) using an Apollo X detector (EDAX Inc., Mahwah, NJ, USA) attached to the SEM, which was operated at an accelerating voltage of 5 kV. The 5 kV accelerating voltage was selected to reduce the electron beam interaction volume when comparing elemental distributions across the carbon fiber, nickel coating, and matrix. For these EDS analyses, specimens were mounted in epoxy resin, polished, and subsequently gold-sputtered to facilitate high-vacuum observation.

## 3. Results

### 3.1. As-Received Reinforcement and As-Fabricated Laminate

**Figure 3:**
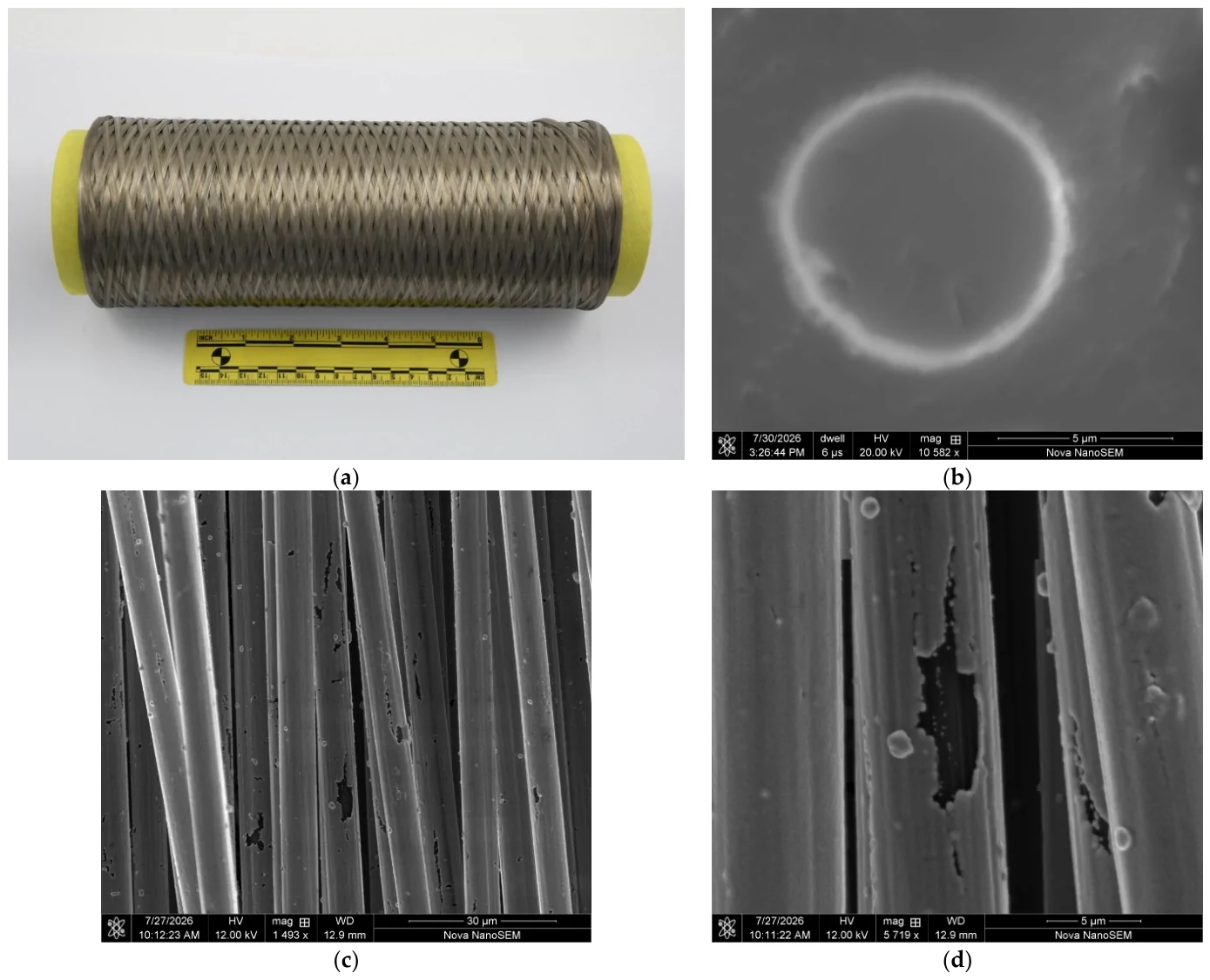
(**a**) Fiber tow in the as-received condition; (**b**) BSE image of polished cross-sections of the as-received coated fibers mounted in epoxy resin; (**c**) top-view SE image showing local dark-contrast features interpreted morphologically as possible coating discontinuities; and (**d**) higher-magnification SE image of the coating-surface morphology.

Figure 3a shows the fiber tow in the as-received condition, while Figure 3b presents SEM images of polished cross-sections mounted in epoxy resin, showing the coating around the carbon fibers. Figure 3c,d show top-view SEM images of the tow surface. The coating was generally uniform in the examined fields, although local dark features were interpreted morphologically as possible coating discontinuities.

The as-fabricated nickel-coated carbon-fiber/geopolymer-derived aluminosilicate laminates had a mean bulk density of 1.94 ± 0.16 g/cm^3^ and a mean apparent porosity of 23.8 ± 2.5% (four coupons from four panels), as summarized in Table 4 and Table 5. The corresponding coefficients of variation were 8.3% and 10.6%, respectively. These measurements describe the bulk physical state of the laminate; apparent porosity is not a direct measure of oxygen diffusivity within the material.

**Figure 4:**
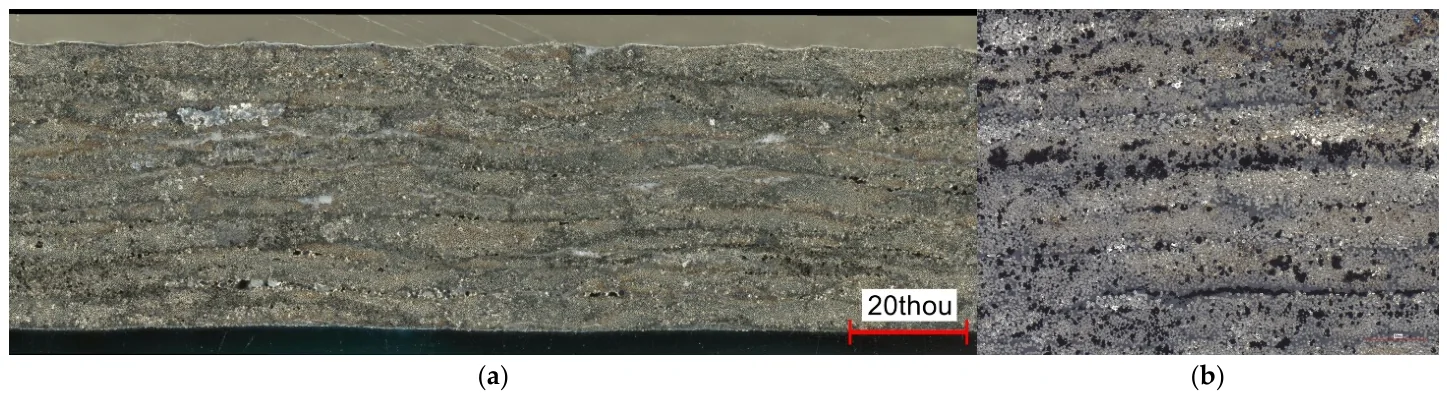
Representative polished cross-sections of the as-fabricated laminate: (**a**) lower-magnification overview showing the distribution of fibers, matrix regions, and dark features; and (**b**) higher-magnification view showing nickel-coated carbon fibers, the surrounding geopolymer-derived matrix, and dark features interpreted morphologically as possible pores in the examined two-dimensional section.

Figure 4a,b present representative polished cross-sectional views of the as-fabricated unidirectional laminate. At lower magnification, dark features interpreted morphologically as possible pores are distributed across the examined cross-section. Under higher magnification, the nickel-coated carbon fibers, matrix phase, and dark pore-like features are visible. These observations are consistent with the apparent porosity measured by immersion. The bulk-density and apparent-porosity data show that the manufactured laminate retained a substantial liquid-accessible pore fraction.

### 3.2. Tensile Properties

Table 6 summarizes the tensile results. For the [0]_8_ coupons, the mean strength was 827.0 ± 31.2 MPa at room temperature, 832.1 ± 94.0 MPa at 371 °C, 745.7 ± 74.7 MPa at 427 °C, and 365.5 ± 39.2 MPa at 538 °C. For the [0/90]_2S_ coupons tested at room temperature, the mean strength was 345.7 ± 20.0 MPa.

At room temperature, the mean tensile modulus was 113.1 ± 1.4 GPa for the [0]_8_ coupons and 57.5 ± 3.4 GPa for the [0/90]_2S_ coupons. For the [0]_8_ coupons, the mean modulus was 103.4 ± 0.7 GPa at 371 °C, 101.4 ± 2.1 GPa at 427 °C, and 93.1 ± 2.8 GPa at 538 °C.

**Figure 5:**
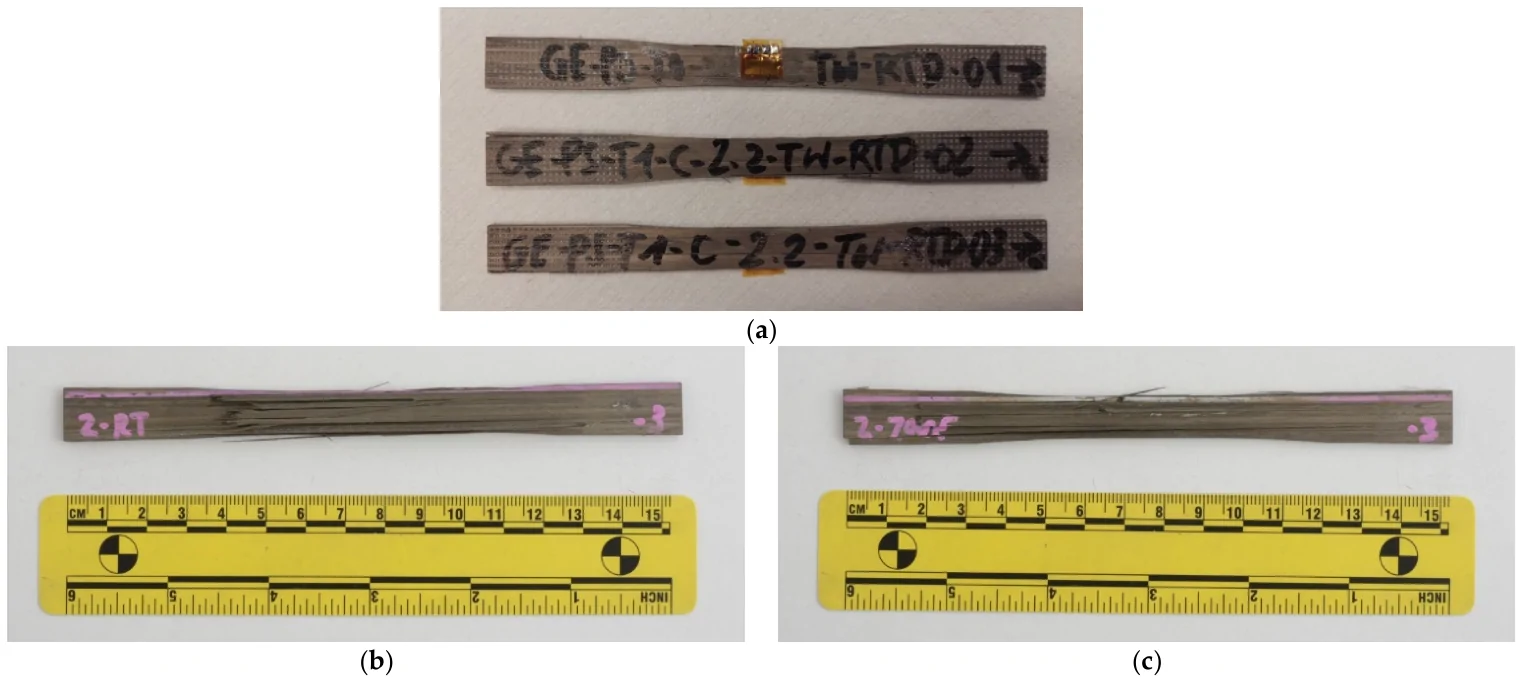

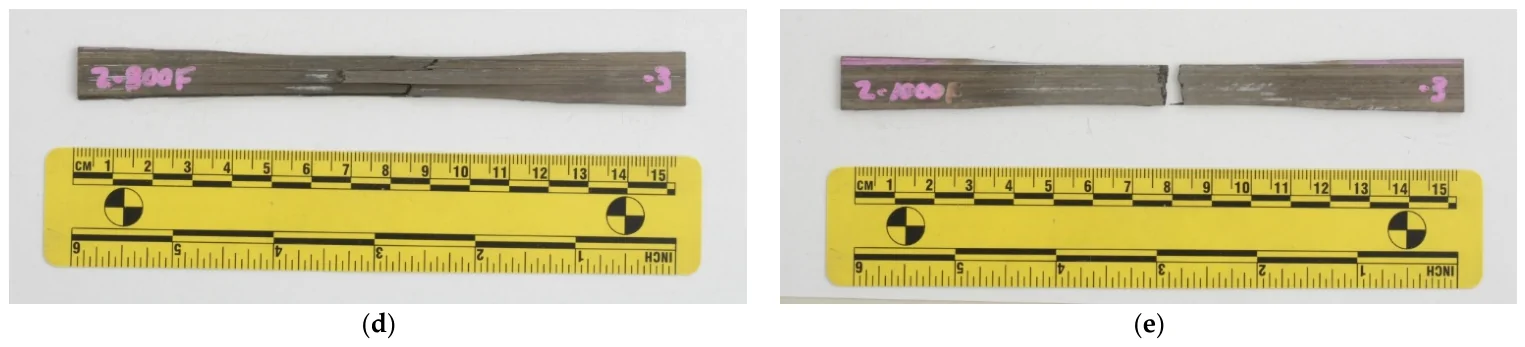
Representative coupons after tensile testing using the procedure described in Section 2.3.2: (**a**) [0/90]_2S_ at RT; (**b**) [0]_8_ at RT; (**c**) [0]_8_ at 371 °C; (**d**) [0]_8_ at 427 °C; and (**e**) [0]_8_ at 538 °C.

The representative [0]_8_ coupons shown in Figure 5 indicate a change in the visible failure mode as a function of temperature. At room temperature and 371 °C, longitudinal splitting was the predominant visible failure mode. A grip-adjacent final fracture was also observed in some coupons that showed longitudinal splitting. At 427 °C, the post-test coupons showed mixed splitting and fiber-dominated tensile fracture within the gauge section. At 538 °C, failure was predominantly brittle and transverse to the fiber direction within the gauge section, without apparent pull-out. The mean strength at 538 °C was 55.8% lower than the room-temperature mean.

**Figure 6:**
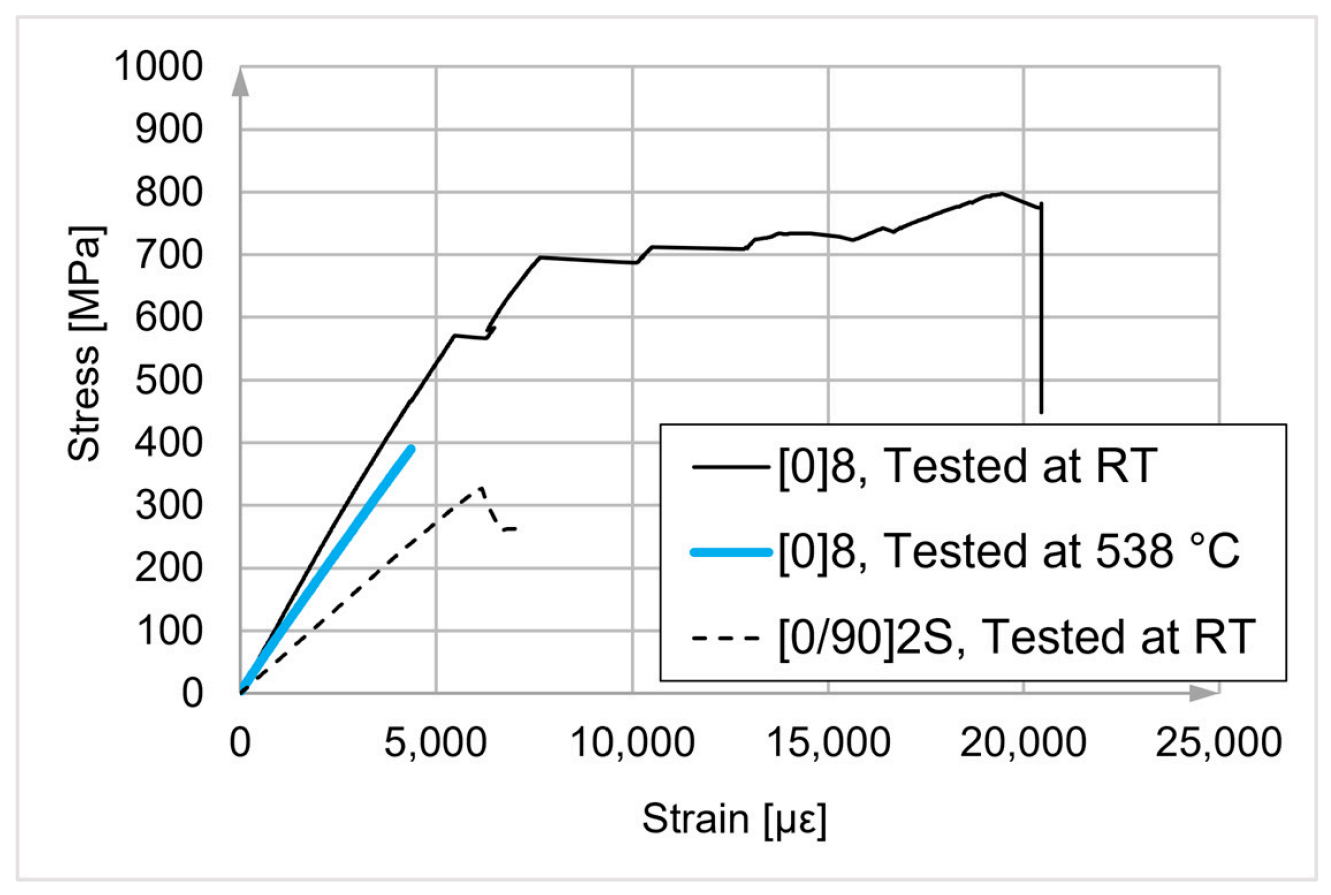
Representative recorded engineering stress–strain curves for the [0]_8_ and [0/90]_2S_ coupons, which used different laminate lay-ups and strain instrumentation. The room-temperature [0]_8_ specimens exhibit the first abrupt strain discontinuity at approximately 5.5–6.5 × 10^3^ µε and 570–580 MPa; this feature may reflect the onset of longitudinal matrix splitting, extensometer slippage, or both. Strain beyond this feature is not interpreted as material strain and is not used to infer failure strain; no precise damage-initiation stress is assigned from the feature.

Extensometer slippage prior to reaching the peak load prevented the acquisition of strain-at-failure data for the unidirectional specimens tested at RT, 371 °C, and 427 °C. For the room-temperature [0]_8_ specimens, the first abrupt strain discontinuity was observed within the approximate ranges of 5.5–6.5 × 10^3^ µε and 570–580 MPa. This feature was consistent with longitudinal matrix splitting, extensometer slippage, or both; it does not identify a precise damage-initiation stress. Valid strain measurements were limited to the [0/90]_2S_ specimens instrumented with strain gauges and the unidirectional specimens tested at 538 °C, which exhibited an approximately linear stress–strain curve up to failure. Representative stress–strain curves for the unidirectional lay-up tested at RT and 538 °C and for the balanced lay-up tested at RT are presented in Figure 6. The RT-tested balanced cross-ply specimens showed predominantly linear behavior, with minor pre-failure non-linearity. The recorded 538 °C curve was approximately linear up to failure.

**Figure 7:**
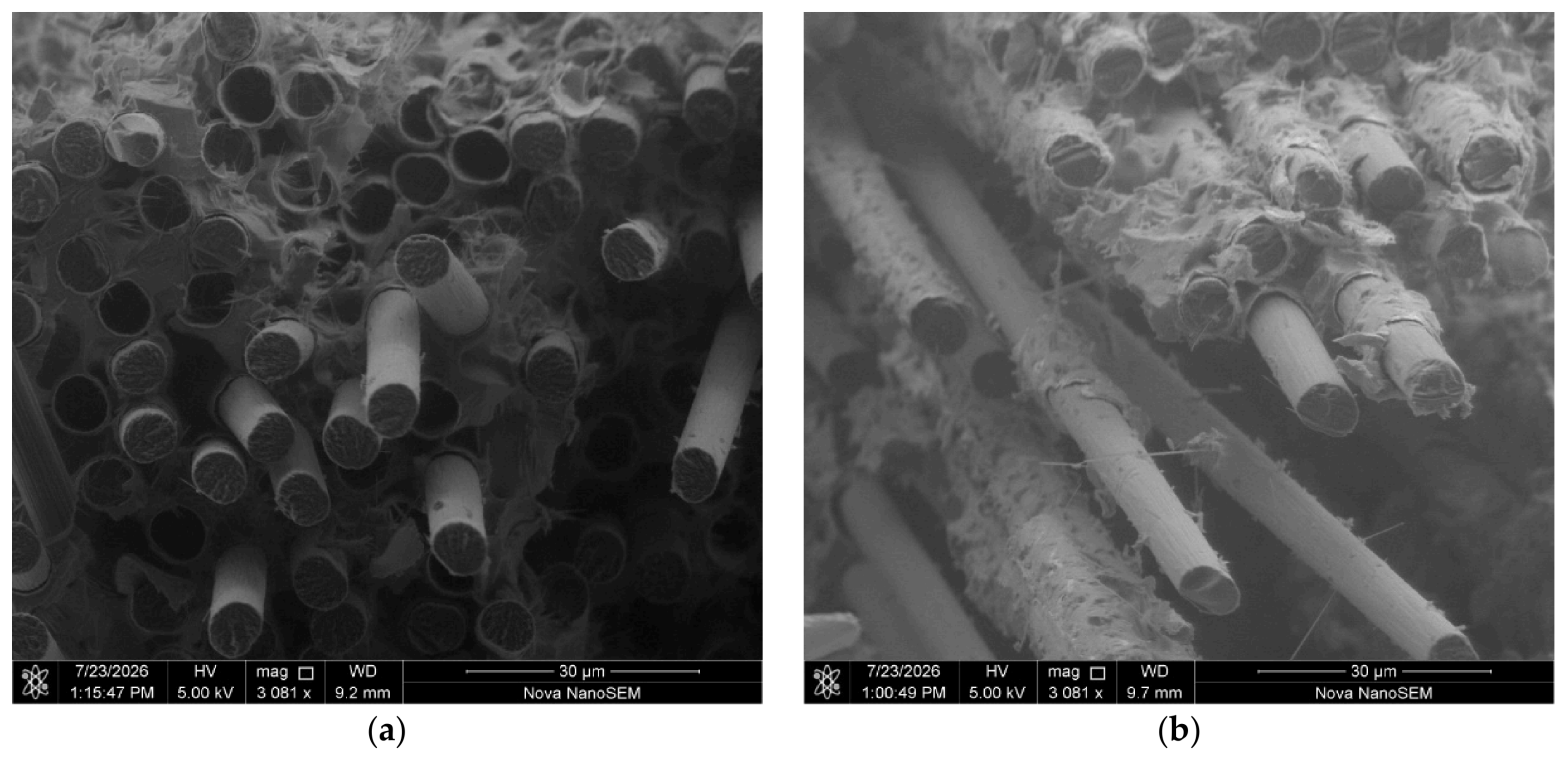

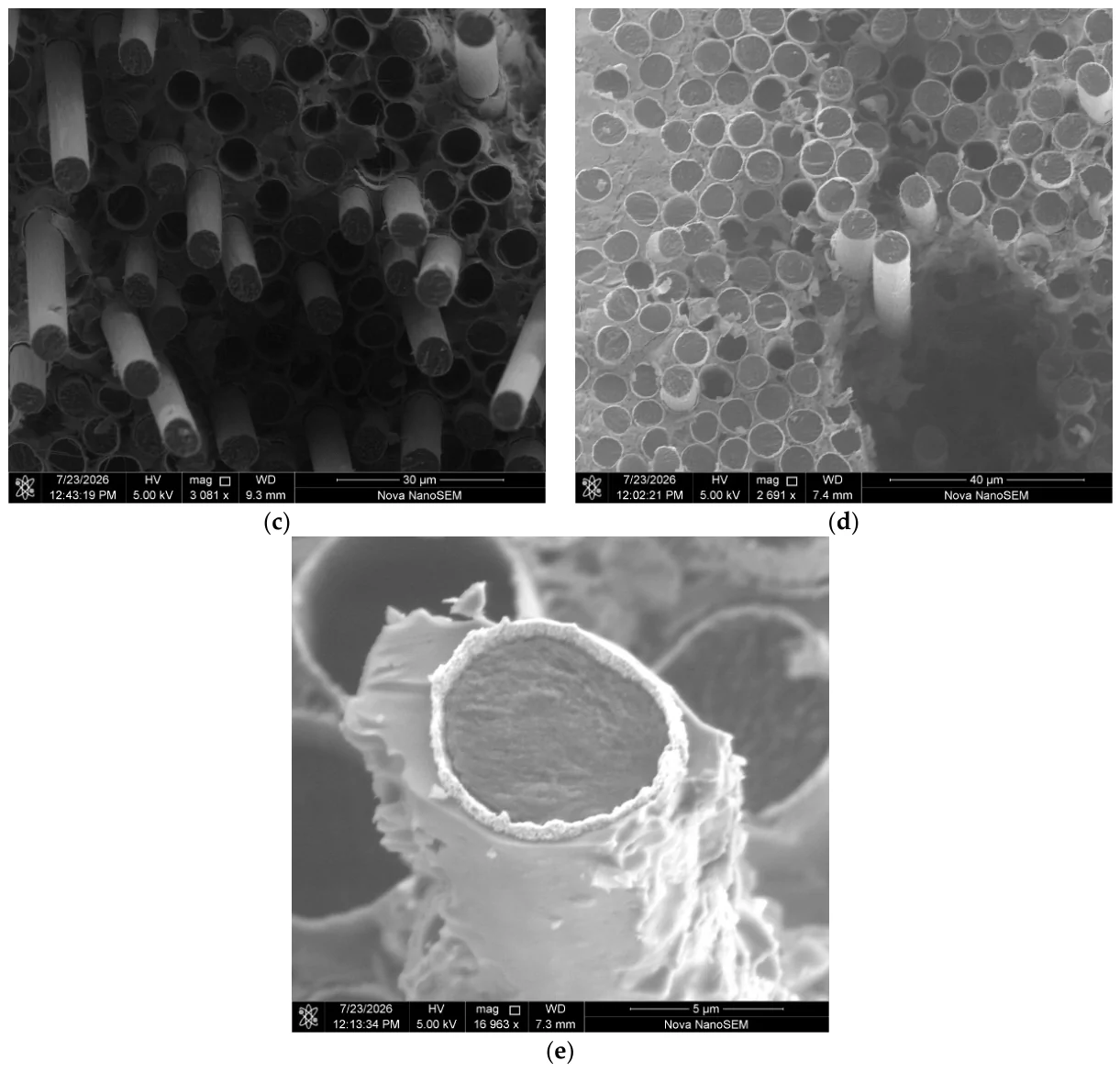
Representative fracture-surface observations from tensile-tested [0]_8_ coupons: (**a**) RT and (**b**) 371 °C, showing loose fibers and pull-out; (**c**) 427 °C, showing transitional fracture morphology; (**d**) 538 °C, showing predominantly transverse filament fracture; and (**e**) 538 °C, showing grain-like coating surface morphology.

Scanning electron microscopy documented representative fracture-surface morphologies associated with the macroscopically observed failure modes and temperature-dependent tensile behavior. Figure 7a–d show extensive loose fibers and fiber pull-out in the specimens tested at RT and 371 °C. Fiber pull-out occurred at the carbon–nickel interface, leaving the nickel coating attached to the surrounding matrix. At higher temperatures, the fracture surfaces became increasingly planar and showed transverse filament fracture, most prominently at 538 °C. In addition, the electrolytic nickel coating exhibited a distinct change in morphology after testing at 538 °C, displaying a pronounced grain-like appearance (Figure 7e) that was not observed on the as-received fiber (Figure 3d). No obvious loss of fiber cross-section attributable to oxidation was observed in the examined 427 °C and 538 °C tensile-fracture micrographs after the short pre-test holds.

**Figure 8:**
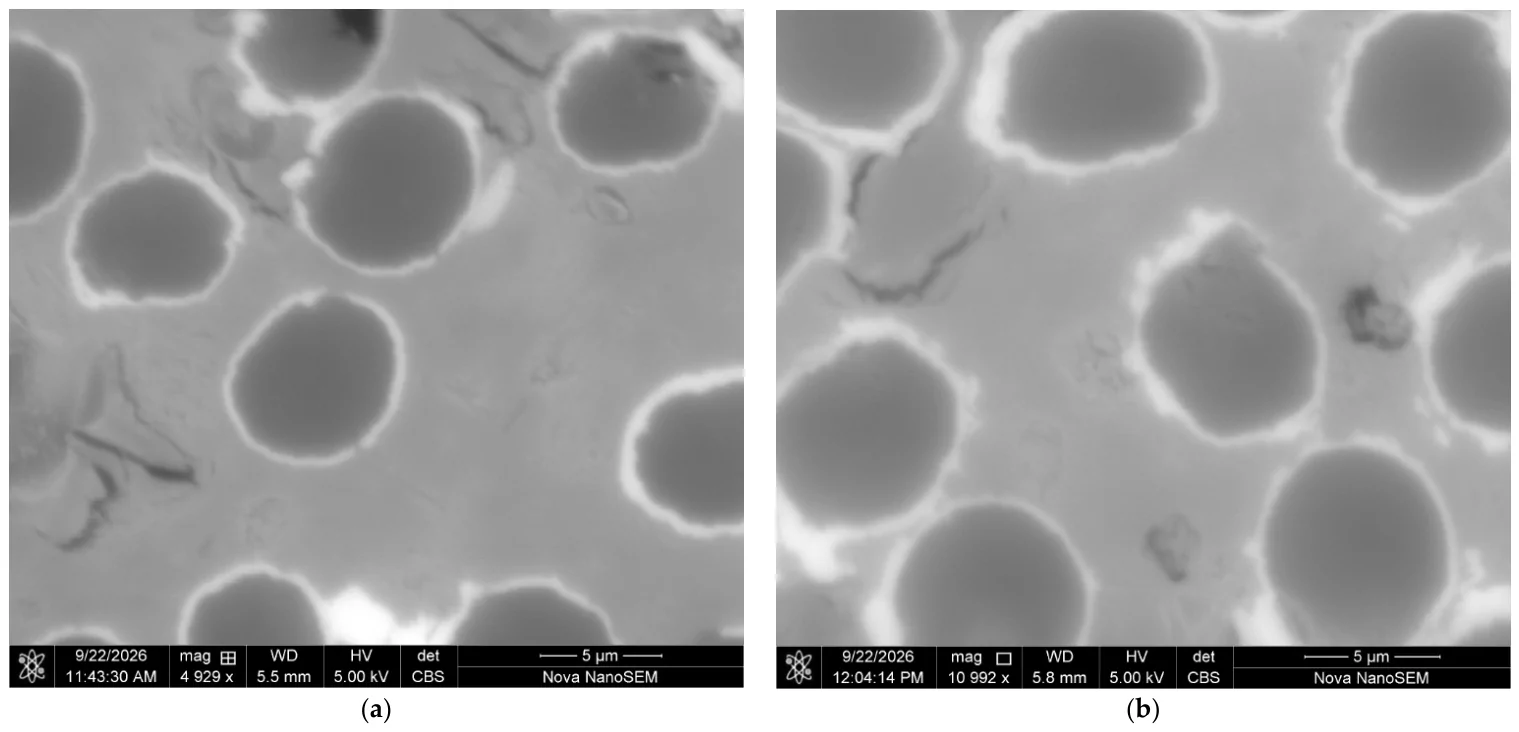

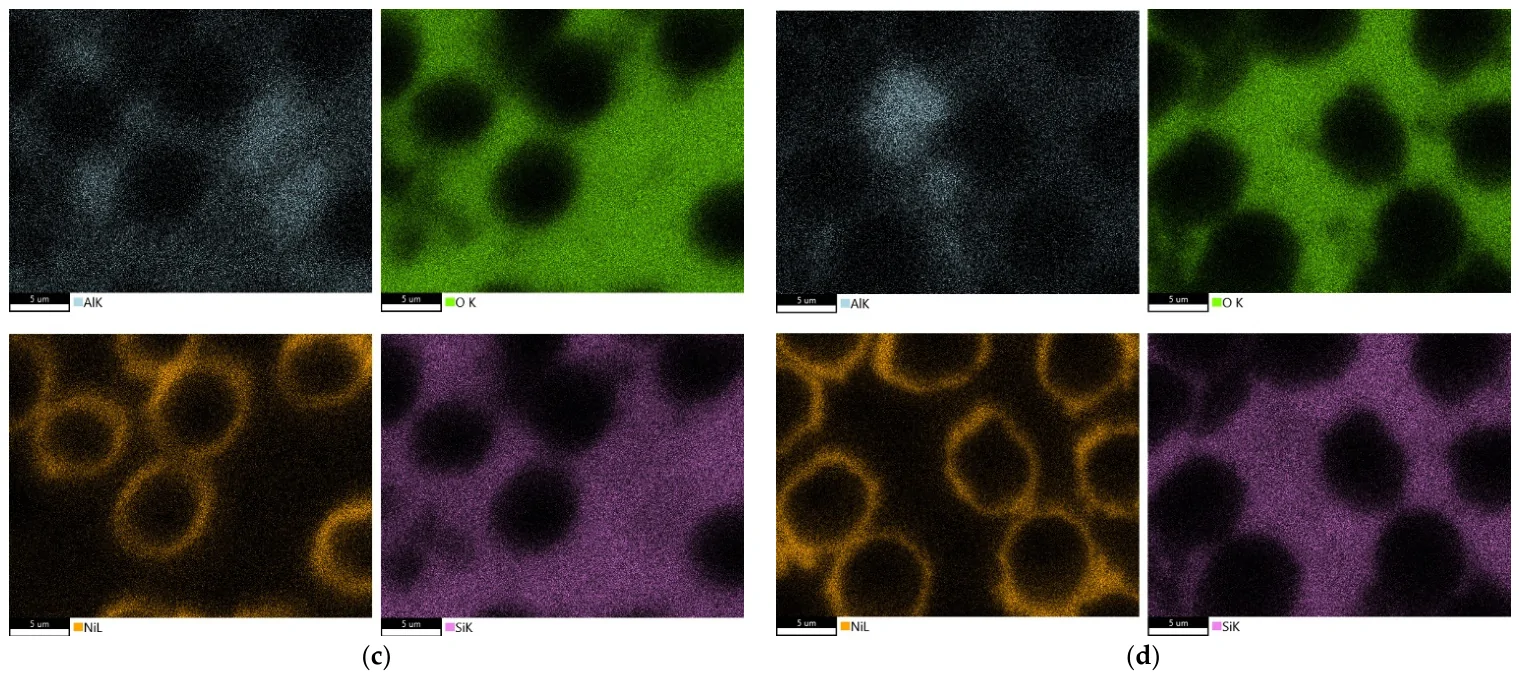
(**a**) BSE-SEM image of representative cross-sections through the gage section of specimen tested in tension at room temperature; (**b**) BSE-SEM image of representative cross-sections through the gage section of specimen tested at 538 °C; (**c**) EDS maps of the location (**a**); (**d**) EDS maps of the location (**b**).

BSE-SEM imaging and EDS mapping of cross-sections from specimens tested at RT and 538 °C showed no distinguishable differences in coating cross-sectional morphology or elemental distributions within the spatial and compositional resolution of the applied methods (Figure 8a–d). The nickel coating remained distinguishable around the carbon fibers and was surrounded by the geopolymer matrix. The observed fracture-mode transition was therefore not accompanied by a resolvable difference in the examined coating cross-sections.

### 3.3. Mass Change and Microstructure After Air Exposure

**Figure 9:**
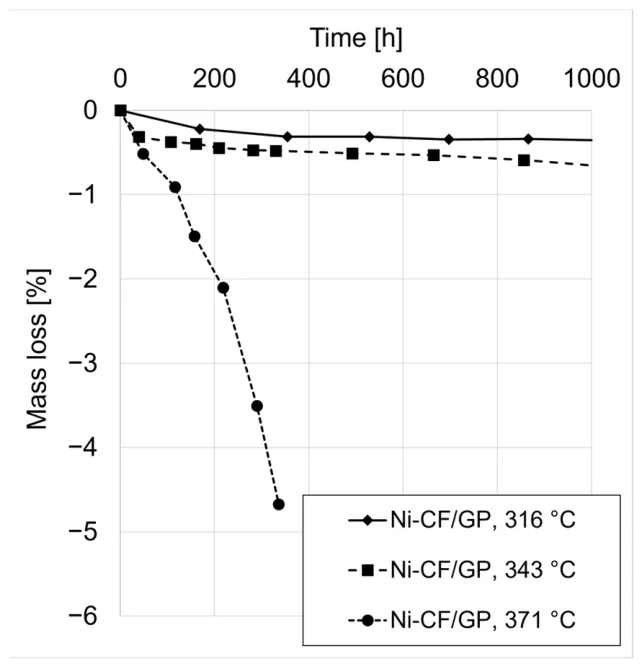
Net mass change in the investigated [0]_8_ laminate during exposure in air at 316, 343, and 371 °C; the 371 °C exposure ended at 336 h. Negative plotted values denote mass loss relative to the initial mass.

To complement the tensile tests after a short dwell at temperature, thermo-oxidative aging was evaluated during prolonged isothermal exposure in air. The exposure temperatures were 316 °C, 343 °C, and 371 °C. The corresponding mass loss as a function of exposure time is shown in Figure 9.

**Figure 10:**
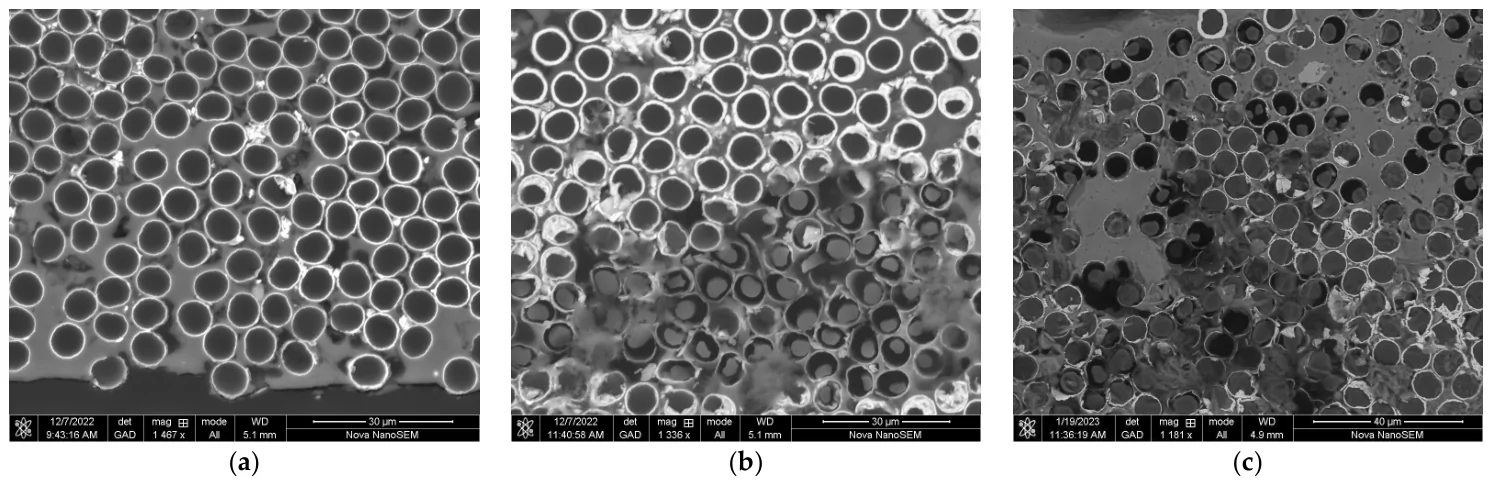
Representative polished cross-sectional SEM images after exposure in air: (**a**) 316 °C for 1000 h, with no obvious filament thinning in the examined field; (**b**) 343 °C for 1000 h, with localized filament thinning; and (**c**) 371 °C for 336 h, with non-uniform degradation, including severe thinning and locations with no visible carbon core.

At 316 °C, a small initial net mass loss of less than 0.4% was recorded during approximately the first 200 h of exposure. After this initial decrease, the measured mass remained essentially unchanged for the remainder of the 1000 h test. Cross-sectional SEM micrographs of specimens exposed at 316 °C for 1000 h are shown in Figure 10a. No obvious filament thinning or other microstructural feature attributable to fiber degradation was observed in the examined cross-sections.

At 343 °C, an initial net mass loss of approximately 0.5% was recorded within the first 50 h of exposure. Beyond this initial stage, the measured net mass loss increased gradually over the remainder of the test. The mass-change curve became slightly steeper after roughly 600 h. SEM observations of specimens exposed at 343 °C for 1000 h are shown in Figure 10b. In these sections, localized reductions in fiber diameter were observed in a limited number of filaments. In the affected fibers, a visible gap was present between the carbon core and the surrounding nickel coating. However, most filaments remained intact, and no consistent pattern in the location of the affected fibers was evident across the section.

**Figure 11:**
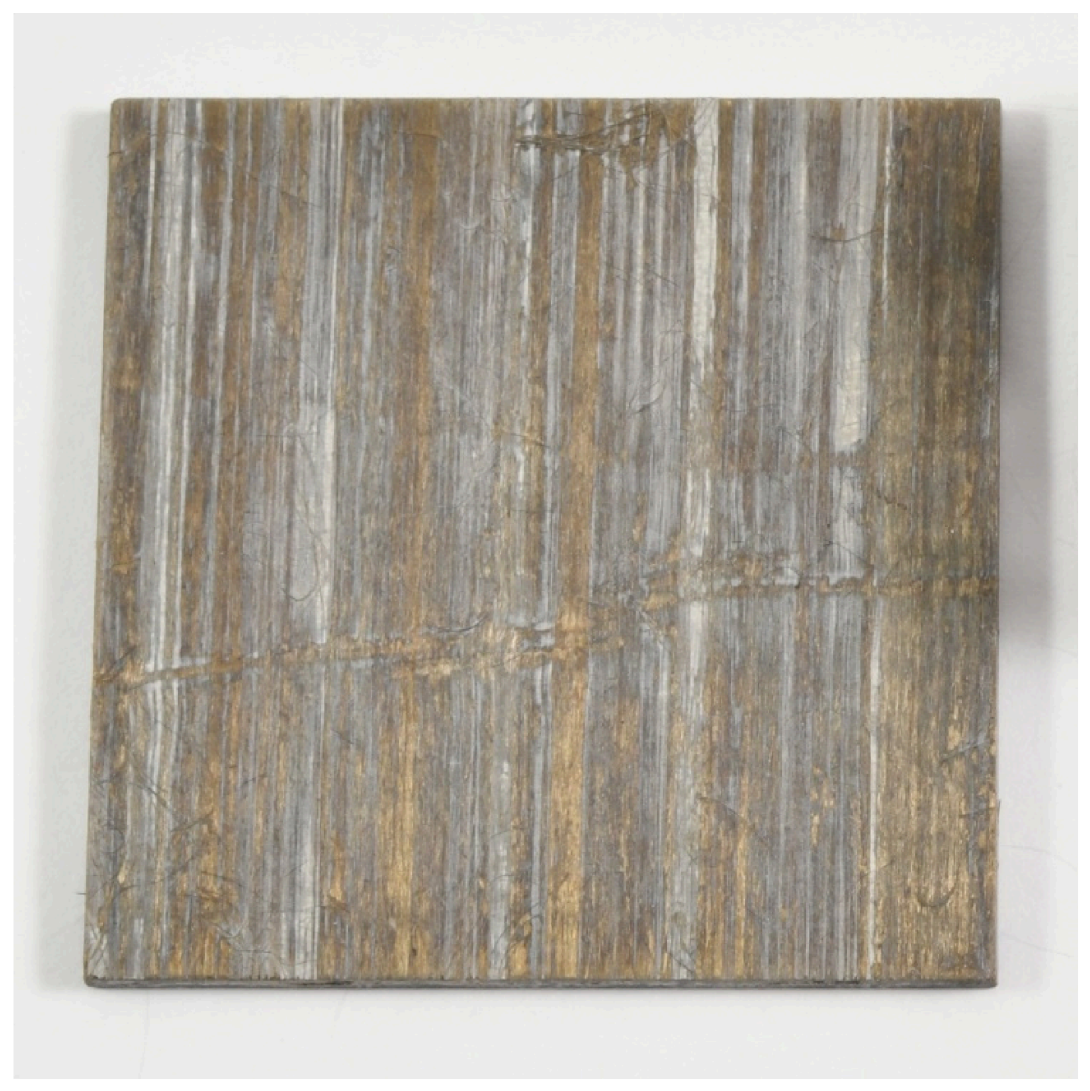
Macroscopic appearance of a representative specimen after exposure to air at 371 °C for 336 h. The observed yellow discoloration is reported descriptively and is not assigned a composition or protective function.

At 371 °C, the measured net mass loss reached approximately 4.7% after 336 h of exposure. The test was stopped at that point so that the specimens could be retained for microstructural characterization. Cross-sectional SEM observations showed extensive fiber degradation, including severely thinned filaments, filaments in intermediate stages of attack, and locations with no visible carbon core. At the same time, a substantial number of filaments showed no obvious reduction in cross-sectional size. A macroscopic image of a representative specimen after oxidation at 371 °C for 336 h is shown in Figure 11. Although the specimen retained its overall shape, the surface exhibited a distinct yellow discoloration after exposure.

Across the investigated temperature–duration combinations, higher temperatures were accompanied by greater net mass loss and more extensive filament degradation. At 316 °C, only a small initial mass loss was recorded, with no obvious filament thinning in the examined sections after 1000 h. At 343 °C, the mass loss remained relatively limited, although localized fiber thinning was observed in a small fraction of the reinforcement. At 371 °C, substantially greater mass loss was measured after a shorter exposure time, and the corresponding micrographs showed extensive, non-uniform filament degradation.

## 4. Discussion

This study establishes initial physical-property, tensile, fracture-morphology, and prolonged air-aging data for continuous nickel-coated carbon-fiber/geopolymer-derived aluminosilicate laminates. Rigid, unidirectional, and balanced cross-ply panels were manufactured. The [0]_8_ mean tensile strength was 827.0 MPa at room temperature and 365.5 MPa at 538 °C. Net mass loss was below 0.4% after 1000 h at 316 °C and approximately 4.7% after 336 h at 371 °C. These measurements meet the feasibility objective but do not establish an operating-temperature range or application readiness.

The measured bulk density was 1.94 ± 0.16 g/cm^3^. This value reflects the densities and proportions of the carbon reinforcement, aluminosilicate matrix, and nickel coating, together with the porosity retained after processing. The Grafil 34-700 carbon fiber has a density of 1.80 g/cm^3^ [43], whereas nickel has a reference density of approximately 8.90 g/cm^3^ [44], making the coating a comparatively dense constituent. The apparent porosity of 23.8 ± 2.5% indicates a substantial liquid-accessible pore fraction that contributes to a lower bulk density than that of a pore-free material of the same solid composition.

Beyond its contribution to bulk density, porosity may influence oxygen access during elevated-temperature exposure. ASTM C20 [41] measures liquid-accessible apparent porosity but does not establish gas permeability or oxygen flux. Studies of transport in heated metakaolin-based geopolymers and oxidation models for carbon/ceramic composites, nevertheless, suggest a possible role for pores in providing access to the reinforcement [33,34,45]. If oxygen reaches the carbon through possible local discontinuities in the nickel coating, fiber oxidative damage could reduce the effective strength of affected fibers and create additional voids, potentially facilitating further oxygen ingress and localized fiber consumption [33,34,35,46,47]. This proposed pathway is a working hypothesis that depends on pore geometry and connectivity, coating continuity, and their evolution during exposure. Whether further consolidation improves load transfer or resistance to oxidation requires controlled evaluation.

For the [0]_8_ specimens, the mean modulus decreased from 113.1 GPa at room temperature to 103.4 GPa at 371 °C, whereas the mean tensile strengths were similar at 827.0 and 832.1 MPa, respectively. A temperature-dependent reduction in matrix stiffness may have contributed to the lower composite modulus by decreasing the matrix contribution to the initial longitudinal stiffness. A reduction in matrix stiffness would not necessarily cause a proportional decrease in ultimate tensile strength, provided that the fibers retain their load-bearing capacity and sufficient load transfer is maintained. At a given composite strain, a lower matrix modulus would also reduce the axial stress carried by the matrix. Whether lower matrix stiffness delays the onset of matrix cracking would also depend on the matrix strength and local stress state. The longitudinal splitting visible in the post-test specimens in Figure 5b,c is consistent with damage within the matrix and/or along constituent interfaces, but it does not establish the location of damage initiation or demonstrate delayed cracking at 371 °C. Increased matrix compliance may therefore have contributed to the decrease in mean tensile modulus, although its magnitude cannot be quantified from the present measurements. Studies of uncoated PAN-based carbon fibers in oxygen-containing environments report little or no tensile-strength degradation at 300 °C and only a slight decrease below 400 °C; Li and Xian also estimated the onset of oxidation at approximately 354 °C [46,47]. Although these results are not directly transferable to the coated fibers, they suggest that 371 °C lies within a transition regime in which exposure duration and oxygen access may increasingly affect fiber properties.

The mean [0]_8_ tensile strength decreased from 832.1 MPa at 371 °C to 745.7 MPa at 427 °C, followed by a pronounced decrease to 365.5 MPa at 538 °C. This reduction was accompanied by a clear evolution in failure morphology. At RT and 371 °C, the specimens failed primarily through longitudinal splitting and fiber pull-out. Separation occurred preferentially at the carbon–nickel interface, leaving the nickel coating attached to the surrounding matrix. At 427 °C, longitudinal splitting was accompanied by fiber-dominated fracture within the gauge section. At 538 °C, the specimens exhibited predominantly brittle fracture transverse to the fiber direction, and fiber pull-out was no longer apparent. Thus, as the measured strength decreased, the dominant failure mode shifted from longitudinal splitting and interfacial separation toward direct fiber fracture.

At RT and 371 °C, carbon filaments were pulled out from the nickel coating, while the coating remained attached to the surrounding matrix. In contrast, pull-out was no longer apparent at 538 °C, where the filaments fractured predominantly in a brittle manner transverse to the fiber direction. This morphology is consistent with filament fracture preceding extensive debonding and sliding at the carbon–nickel interface. One possible explanation is a reduction in filament strength caused by localized carbon oxidation. If liquid-accessible pores are connected under the exposure conditions, they could shorten oxygen-transport paths to possible local coating discontinuities, where oxidation could reduce the strength of individual filaments [46,47]. Failure of locally weakened filaments would transfer additional load to neighboring fibers and could promote fracture across a larger group of filaments. Alternatively, a change in interfacial properties at the carbon–nickel interface could alter the competition between filament fracture and pull-out. Overall, the observed morphology indicates a shift from an interface-mediated pull-out mechanism toward fiber-dominated fracture, potentially resulting from fiber weakening, a change in interfacial sliding resistance, or both. The associated reduction in interfacial sliding and pull-out would limit frictional energy dissipation [2], consistent with the more brittle failure observed at 538 °C.

The fracture-surface observations provide additional information on the possible contribution of the nickel coating to the temperature-dependent change in failure mode. After testing at 538 °C, the coating exhibited a distinct grain-like surface appearance that was not observed on the as-received fibers. Localized plastic deformation of the nickel coating was nevertheless visible in some of the examined filament fractures. The coexistence of a grain-like surface appearance and local plastic deformation suggests that uniform embrittlement of the coating alone is unlikely to account for the transition from carbon–nickel interfacial pull-out at lower temperatures to predominantly transverse filament fracture at 538 °C.

BSE-SEM images and corresponding EDS maps were obtained from polished cross-sections of specimens tested at RT and 538 °C. No temperature-associated difference in coating cross-sectional morphology or elemental distributions was distinguishable within the spatial and compositional resolution of the applied methods. These observations do not conflict with the fracture-surface morphology because the two analyses represent different microstructural features: the fracture surfaces reveal local deformation and separation processes, whereas the polished cross-sections show the retained coating geometry and elemental distribution away from the immediate fracture path. The grain-like appearance may therefore reflect deformation-induced surface relief, surface oxidation, thermally induced evolution of the nickel microstructure, or a combination of these effects; morphology alone does not demonstrate grain growth or recrystallization.

Thermally induced evolution of the coating, nevertheless, remains a plausible contributor. Annealing studies of electrodeposited nickel have demonstrated that grain growth and recrystallization can occur over the temperature range relevant to the present tests, although their onset and extent depend strongly on the initial grain size, texture, impurity content, and deposition conditions [48]. If comparable microstructural evolution occurred in the present coating, it could, together with thermal-expansion mismatch among the carbon fibers, nickel coating, and matrix, modify local residual stresses and the resistance to debonding or sliding at the carbon–nickel interface. This could contribute to the suppression of fiber pull-out and increase the likelihood of direct filament fracture, but the present observations do not clearly establish this mechanism.

To distinguish among these possible mechanisms, high-temperature DIC combined with targeted fractography could relate strain localization and longitudinal splitting to the observed failure modes while distinguishing specimen deformation from extensometer slippage. Comparative crystallographic characterization could test whether the grain-like appearance reflects grain evolution, while targeted chemical and interfacial measurements could examine changes in debonding and sliding resistance.

The air-exposure results reveal strongly non-uniform filament degradation. At 316 °C, net mass loss remained below 0.4% after 1000 h, with no obvious filament thinning in the examined sections. Localized thinning was observed after 1000 h at 343 °C, whereas after 336 h at 371 °C, neighboring filament cross-sections ranged from apparently intact to severely thinned or lacking a visible carbon core. Carbon oxidation is thermodynamically favorable over this temperature range [35]; its local rate and extent, however, depend on oxygen access and the effectiveness of the surrounding barrier. A study of electroless Ni–P-coated carbon fibers, although chemically distinct from the present electrolytic nickel coating, illustrates how coating chemistry and thermal evolution can affect oxidation behavior [36]. In the present laminate, possible discontinuities in the as-received coating, liquid-accessible pores, local differences in matrix coverage and fiber–matrix contact, and geometric shielding by the surrounding matrix and adjacent fibers are plausible contributors to the non-uniform filament degradation. These observations are consistent with the working hypothesis that local differences in barrier continuity and oxygen accessibility contribute to selective filament consumption.

PMR-15 provides a practical reference for interpreting the present study results. The NASA sources show that the mass change in T650-35/PMR-15 during air aging depends on specimen thickness and exposed-edge fraction [16,49]. The disused eight-ply specimens were produced from T650-35 eight-harness satin woven fabric and measured approximately 89.4 × 108.3 × 2.5 mm, with a reported cut-edge-to-total-surface-area ratio of 5.4% [49]. In comparison, the present unidirectional [0]_8_ coupons measured 40 × 40 × 2.1 mm and had a calculated cut-edge-to-total-surface-area ratio of 9.50%. While the published T650-35/PMR-15 curve indicates approximately 3.6% mass loss after 1000 h in air at 316 °C [16], the present laminate exhibited less than 0.4% net mass loss under the same nominal temperature and exposure duration, with no obvious filament thinning in the examined sections. The present laminate showed a lower net mass loss in this qualitative comparison. Because the measured mass change may combine carbon loss with mass gain associated with nickel oxidation, the mass data are considered together with the corresponding microscopy. Differences in reinforcement form, laminate lay-up, specimen dimensions, composition, and exposed-fiber geometry preclude a direct quantitative ranking. Nevertheless, the combination of low net mass loss and the absence of obvious filament thinning supports continued evaluation of the present material for prolonged exposure at 316 °C. At higher temperatures, localized filament thinning was observed after 1000 h at 343 °C, while approximately 4.7% net mass loss and severe, non-uniform filament degradation occurred after 336 h at 371 °C. These observations indicate increasing susceptibility to localized carbon oxidation with increasing exposure temperature, although the present results do not establish whether this arises primarily from oxygen transport through the matrix, possible local discontinuities in the nickel coating, changes in the coating during prolonged exposure, or a combination of these mechanisms. The literature nevertheless provides a relevant basis for considering coating continuity during further material development.

For electroplated T300 carbon fibers, Lu et al. [50] found that a small amount of deposited nickel, insufficient to provide effective surface coverage, accelerated carbon oxidation, whereas a larger deposit that more fully covered the fiber surface improved oxidation resistance. Their results show that the effect of nickel depends on the extent and continuity of surface protection rather than on the presence of nickel alone. Studies of electroless Ni–P-coated carbon fibers likewise showed that nickel-containing coatings can delay the onset of fiber oxidation [36], although the coating chemistry and thermal transformations reported for Ni–P cannot be directly transferred to the electrolytic nickel coating used here. The present results do not isolate the contribution of nickel to oxidation resistance. Together with the cited studies, they motivate examining how coating continuity affects oxidation during prolonged exposure above 316 °C. The present study does not determine an optimum nickel content, coating thickness, or coverage.

Table 7 presents the current mechanical and prolonged air-exposure results alongside published data for continuous-carbon-fiber/geopolymer composites and the selected T650-35/PMR-15 reference. The reinforcement form, laminate lay-up, test type, loading direction, and thermal history reported for each material are retained. Differences in fiber content, constituent properties, consolidation, fabric crimp, and interfacial load transfer preclude a quantitative comparison of fiber load-transfer efficiency across the materials. The comparisons, therefore, remain qualitative. Published flexural values are identified separately and are not ranked against the present tensile measurements.

The manufactured panels, tensile properties, and low net mass change during prolonged air exposure at 316 °C justify continued evaluation of this continuous-fiber composite material. The remaining questions are whether improved consolidation and lower liquid-accessible porosity reduce oxygen access, how nickel-coating continuity affects local carbon oxidation, and how the carbon–nickel interface changes with processing, exposure environment, temperature, and duration.

## 5. Conclusions

1. Rigid unidirectional and balanced cross-ply laminates comprising nickel-coated carbon fibers and a geopolymer-derived aluminosilicate matrix were manufactured. They had a bulk density of 1.94 ± 0.16 g/cm^3^, an estimated reinforcement volume fraction of 45–51%, and an apparent porosity of 23.8 ± 2.5%.
2. The mean [0]_8_ tensile strengths were 827.0 ± 31.2 MPa at room temperature, 832.1 ± 94.0 MPa at 371 °C, 745.7 ± 74.7 MPa at 427 °C, and 365.5 ± 39.2 MPa at 538 °C. The room-temperature and 371 °C mean tensile strengths were similar, while the mean modulus decreased from 113.1 to 103.4 GPa. The mean [0/90]_2S_ tensile strength was 345.7 ± 20.0 MPa. Comparisons with published woven laminates remain qualitative as reinforcement form, lay-up, constituents, and test conditions differ.
3. Failure remained dominated by longitudinal splitting and carbon–nickel interfacial pull-out through 371 °C. Mixed splitting and fiber-dominated gauge-section fracture occurred at 427 °C, followed by predominantly brittle transverse filament fracture without apparent pull-out at 538 °C. No differences in coating cross-sectional morphology or elemental distributions between the room-temperature and 538 °C specimens could be distinguished within the spatial and compositional resolution of the BSE-SEM and EDS methods.
4. After thermo-oxidative aging in air, net mass loss remained below 0.4% at 316 °C after 1000 h and reached approximately 4.7% at 371 °C after 336 h. The examined sections showed no obvious filament thinning at 316 °C, localized thinning after 1000 h at 343 °C, and severe, non-uniform filament degradation at 371 °C. The observed non-uniform filament degradation is consistent with potential local differences in oxygen access associated with possible local coating discontinuities, matrix coverage, and accessible porosity.
5. The net mass loss below 0.4% after 1000 h at 316 °C compares favorably with the approximately 3.6% value estimated from the selected T650-35/PMR-15 curve. This comparison remains qualitative because the reinforcement form, specimen geometry, and constituent chemistry differ.
6. The similar mean [0]_8_ tensile strengths at room temperature and 371 °C, together with splitting- and pull-out-dominated failure through 371 °C and the limited degradation observed after prolonged aging at 316 °C, justify continued evaluation of the material. The fracture-mode transitions at 427 and 538 °C and non-uniform filament degradation during higher-temperature aging show that short-duration tensile behavior and prolonged oxidation resistance require separate assessment.
7. Determining whether the low net mass loss and absence of obvious filament thinning in the examined sections at 316 °C correspond to retained mechanical properties requires mechanical testing after prolonged aging. High-temperature DIC could distinguish specimen strain localization and longitudinal splitting from apparent strain caused by extensometer slippage.
8. The extent to which the nickel coating limits carbon oxidation, and how this effect depends on coating continuity and oxygen accessibility, remain unresolved. Matched laminates reinforced with coated and uncoated fibers would isolate the effect of the coating. Gas-transport measurements and three-dimensional pore characterization could test how connected porosity contributes to oxygen access. Targeted crystallographic and interfacial analyses could determine whether the grain-like surface morphology of the nickel coating at 538 °C reflects grain evolution and whether changes at the carbon–nickel interface contribute to the failure mode transition.

## Figures and Tables

**Table 3:** Summary of the mechanical test campaign, laminate lay-ups, specimen types, and measured properties for nickel-coated carbon-fiber/geopolymer-derived aluminosilicate-matrix composites.

Test	Standard/Method	Number of Specimens	Specimen Condition	Test Temperature	Laminate Lay-Up	Specimen Type and Size	Properties Characterized
Tension, unidirectional laminate	See Section 2.3.2	3	As-fabricated	RT, 371 °C, 427 °C, and 538 °C	[0]_8_	Contoured, face-loaded specimen based on ASTM C1359-18e1, Fig. X2.3; typical thickness approximately 2.1 mm.	Tensile modulus (*E^t^*), tensile strength (*F^tu^*), and strain at failure (*ε^f^*) where a valid strain record was retained
Tension, balanced laminate	See Section 2.3.2	3	As-fabricated	RT	[0/90]_2S_	Contoured, face-loaded specimen based on ASTM C1359-18e1, Fig. X2.3; typical thickness approximately 2.1 mm.	Tensile modulus (*E^t^*), tensile strength (*F^tu^*), and strain at failure (*ε^f^*)

**Table 4:** Bulk density measured by the immersion method described in Section 2.3.1.

Statistic	Value	Unit
*n* (coupons)	4	—
Mean	1.94	g/cm^3^
Standard deviation (SD)	0.16	g/cm^3^
Coefficient of variation (CoV) (%)	8.28	%

**Table 5:** Apparent porosity measured by the immersion method described in Section 2.3.1.

Statistic	Value	Unit
*n* (coupons)	4	—
Mean	23.8	%
Standard deviation (SD)	2.5	%
Coefficient of variation (CoV) (%)	10.6	%

**Table 6:** Tensile properties of the [0/90]_2S_ and [0]_8_ laminates. Values are mean ± SD, with CoV in parentheses; *n* = 3 within-panel coupon replicates per condition.

Laminate	Temperature	Tensile Strength, Ftu (MPa)	Tensile Modulus, *E^t^* (GPa)	Strain at Failure, *ε^f^* (%)
[0/90]_2S_	RT	345.7 ± 20.0 (5.8%)	57.5 ± 3.4 (5.9%)	0.67 ± 0.04 (5.4%)
[0]_8_	RT	827.0 ± 31.2 (3.8%)	113.1 ± 1.4 (1.2%)	—
[0]_8_	371 °C	832.1 ± 94.0 (11.3%)	103.4 ± 0.7 (0.7%)	—
[0]_8_	427 °C	745.7 ± 74.7 (10.0%)	101.4 ± 2.1 (2.1%)	—
[0]_8_	538 °C	365.5 ± 39.2 (10.7%)	93.1 ± 2.8 (3.0%)	0.40 ± 0.06 (14.2%)

Note: Each mean, SD, and CoV is based on three within-panel coupon replicates. CoV values were calculated from the unrounded specimen data and are reported to one decimal place. Failure strain is reported only where a valid strain record was obtained. It is not reported for the [0]_8_ tests at RT, 371 °C, or 427 °C due to extensometer slippage before peak load.

**Table 7:** Mechanical properties and air-aging data for the present material, published continuous-carbon-fiber/geopolymer composites, and the T650-35/PMR-15 polyimide reference.

Test Type	Material/Source	Lay-Up and Property	Test Temperature, Exposure Conditions	Key Result and Comparison Notes
Tension	Present material	Continuous UD [0]_8_; tension along the fiber direction	RT, 371, 427, and 538 °C; elevated-temperature tests followed a 15–30 min hold	827.0 ± 31.2; 832.1 ± 94.0; 745.7 ± 74.7; and 365.5 ± 39.2 MPa, respectively. Present study; all coupons for a condition were machined from one panel.
Tension	Present material	Balanced [0/90]_2S_ cross-ply; tension	RT	345.7 ± 20.0 MPa. Different laminate lay-up and strain instrumentation from the [0]_8_ specimens.
Tension	Lyon et al. [29]	Carbon-fabric cross-ply; tension	RT	343 ± 31 MPa. Woven cross-ply laminate; not a matched control for the present laminates.
Flexure	Lyon et al. [29]	Carbon-fabric cross-ply; three-point flexure	RT; after more than 60 min in air at 400 °C	245 ± 8 MPa; 163 ± 6 MPa after exposure. Flexural result; no tensile-retention data.
Flexure	Tran et al. [30]	Continuous UD carbon fiber; three-point flexure	RT after curing and drying at 75 °C	Approximately 571 MPa. Different matrix, fiber fraction, test method, and processing.
Tension	Medri et al. [31]	Ten alternating 0/90 carbon-fabric plies; tension	RT after processing stabilization at 750 °C in argon	136.7 ± 3.4 MPa. Lay-up, fiber fraction, and processing differ.
Tension	PyroKarb [40]	Two-dimensional 0/90 carbon fabric; tension	RT and 350 °C	270 ± 25 and 250 ± 25 MPa. Product datasheet; duration and atmosphere not stated.
Tension	T650-35/PMR-15 [38]	Balanced 0/90 ten-ply eight-harness satin woven laminate; tension	22 and 316 °C	855 ± 17.0 and 831 ± 14.3 MPa. Balanced woven laminate; not a direct quantitative comparator for the present unidirectional [0]_8_ tensile data.
Air aging	Present material	Continuous UD [0]_8_; net mass change and selected-field microscopy	316 °C for 1000 h; 371 °C for 336 h in air	Mass loss below 0.4% and approximately 4.7%, respectively.
Air aging	T650-35/PMR-15 [16,49]	8-ply eight-harness satin woven laminate; mass change	316 °C for approximately 1000 h in air	Approximately 3.6% mass loss at 1000 h, estimated from the published graph. Reinforcement form, specimen geometry, exposed-edge fraction, and constituent chemistry differ; qualitative context only.

## Data Availability

The datasets presented in this article are not readily available as they are proprietary and part of an ongoing development program. Requests to access the datasets should be directed to Michal Jasiczek.

## Abbreviations

The following abbreviations are used in this manuscript:

PMC	Polymer-matrix composite
CMC	Ceramic-matrix composite
BMI	Bismaleimide
SEM	Scanning electron microscopy
SD	Standard deviation
CoV	Coefficient of variation
*E^t^*	Tensile modulus
*F^tu^*	Tensile strength
*ε^f^*	Strain at failure
[0]_8_	Unidirectional laminate lay-up with eight 0° plies
[0/90]_2S_	Balanced cross-ply laminate lay-up with symmetric stacking sequence
BSE	Backscattered electron
SE	Secondary electron
EDS	Energy-dispersive X-ray spectroscopy
DIC	Digital image correlation
UD	Unidirectional
RT	Room temperature
*T_g_*	Glass-transition temperature
XPS	X-ray photoelectron spectroscopy
XRD	X-ray diffraction
